# Woman’s Dress

**Published:** 1890-12

**Authors:** 


### WOMAN'S DRESS.

We extract the following experiences of a young lady in search of comfort and convenience in dress, from one of our exchanges:

I have always been a rebel against skirts, and there was war all through my girlhood between my mother and myself over every inch she added to their length. It makes my head ache to think of all the petticoats I used to carry around—flannel, muslin, lace, and the rest—and of all the mud that would accumulate on their dripping flounces, and the big laundry bills. At last I grew desperate, and vowed never to wear another white skirt. Then came the balmoral epoch. Worse still. They didn't show the dirt, which was rather a disadvantage, for they were seldom or never washed in consequence, and that is any thing but desirable in a garment you wear, even if it isn't next to you. And, oh dear! how they did wind themselves about my legs in a high wind, so I could scarcely walk, and tripped and slipped into gutters and pools when I proposed to step over them! So I made up my mind that balmorals must go. Then I considered the Jenness-Miller divided skirt, but made up my mind it was only a makeshift after all, and would go flipperty-flop about my ankles, just the same; so I skipped the divided-skirt stage and leaped at once to freedom, both literally and figuratively, in knickerbockers. Women talk about their emancipation and right of franchise, and quarrel for place on school boards and civil offices, when they might have a kind of liberty in the pursuit of happiness which none or all of all these would ever convey, by gathering their petticoats into two rubber bands at the bottom. I am dressed more warmly, as well as comfortably, than ever before, in a wool combination suit of underclothing, black cashmere knickerbockers, pleated smoothly into a deep, tight yoke at the waist, and, best of all, with actually two pockets in them that I won't lose things out of. Of course, I am apt to retire when I investigate the contents of the pockets, but if I were caught at it I hardly think I'd make a much worse exhibition of myself than does the average woman whirling around after the pocket she can't find, like a pussy cat after her own tail.

